# Diabetic Complications: Current Challenges and Opportunities

**DOI:** 10.1007/s12265-012-9388-1

**Published:** 2012-06-30

**Authors:** Helen D. Nickerson, Sanjoy Dutta

**Affiliations:** JDRF, 26 Broadway, New York, NY 10004 USA

The International Diabetes Federation estimates that 366 million people had diabetes in 2011, and that by 2030, this figure will have risen to a staggering 552 million worldwide. In 2011, diabetes was the cause of 4.6 million deaths and accounted for 11 % of adult healthcare expenditure in the USA [1]. The increasing incidence of both type 1 diabetes (T1D) and type 2 diabetes (T2D) elevates the complications of diabetes as one of the most important current public health issues. Complications of diabetes range from acute, life-threatening conditions such as severe hypoglycemia or ketoacidosis to chronic, debilitating complications affecting multiple organ systems, such as retinopathy, nephropathy, neuropathy, and cardiovascular disease. Estimates of the prevalence of diabetic complications are challenging, in part because there are no internationally agreed upon standards for diagnosis. However, a vast majority of those with diabetes will experience one of more of these complications of diabetes. For example, a recent analysis by the META-EYE study group reported that 93 million people worldwide suffer from diabetic retinopathy. For those with 20 or more years of diabetes, three quarters have some form of diabetic retinopathy [2].

This special issue focuses on progress and challenges in basic and clinical research on these chronic complications of diabetes. The end-stage consequences of diabetic complications can include severe vision loss; end-stage renal disease necessitating dialysis or transplant; myocardial infarction and stroke; and amputations. Many of these life-threatening or disabling events can be preventable with proper “life-long” diabetes care and a healthy lifestyle. The risk of complications is linked to the duration of diabetes and the degree of glycemic control achieved, most commonly assessed by measuring glycated hemoglobin, or HbA1c, a measure of glycemic control over a course of 2 to 3 months. It should be noted that susceptibility to complications varies among individuals with diabetes, and in some cases, complications develop even in those with lower HbA1c values, ostensibly reflecting good glycemic control.

Cardiovascular disease (CVD) is a major cause of mortality in diabetes. In addition to an increased incidence of CVD compared to the general population, people with CVD and diabetes fare more poorly compared to those without diabetes [3]. The prevalence of cardiovascular risk factors in young people with diabetes is of particular concern [4]. The worldwide increase in obesity not only contributes to the increase of T2D, but also exacerbates overall risk of complications in those with T1D, perhaps due to increases in obesity-induced insulin resistance and its pro-inflammatory effects. Therefore, a better understanding of the role of insulin resistance in T1D may offer improved therapeutic approaches and the potential to reduce long-term complications. Cardiac autonomic neuropathy is understudied but may be critical in understanding cardiac events in those with diabetes and may potentially reveal new targets for therapy to prevent and treat CVD in diabetes [5].

The incidence of heart failure is increased in those with diabetes, even in the absence of coronary artery disease, thus additional strategies for treatment and prevention are urgently needed [3]. Microvascular complications of diabetes including diabetic nephropathy, retinopathy, and neuropathy can lead to death and disability.

Although persistent hyperglycemia is acknowledged as a primary driver of diabetic complications, it is not the only factor. Many of these complications are exacerbated by other co-pathologies commonly occurring with diabetes, particularly high blood pressure and lipid abnormalities. Hence, the current clinical approaches that aim to prevent diabetic complications are focused on achieving targets for glycemic, blood pressure, and lipid control [6]. Better glycemic control is having a positive impact on the incidence and progression of microvascular complications in T1D and T2D, though the picture is not so clear for cardiovascular complications, particularly in higher risk subjects with T2D [7]. Further improvements in glucose control should help lower the barrier to achieving glycemic targets and thus reduce complications. For example, recent pivotal trials have suggested benefit for new continuous glucose monitoring approaches in reducing HbA1c levels [8]. Even with the improvements in glycemic control achievable today, the risk of diabetic complications is unacceptably high, perhaps due to individual genetic predisposition. In order to reduce the prevalence of diabetic complications, better approaches to achieve glycemic control and identification of additional targets in diabetes are essential. These may include novel technologies, better education, and for T1D patients, considering individualized incorporation of other glucose-lowering therapies, such as GLP-1, metformin, and other therapies that are currently in clinical trial evaluations.

In addition to hyperglycemia, some researchers have proposed that overall blood glucose variability may represent a risk factor for diabetes complications not completely captured by measurement of HbA1C [9, 10]. This is an intriguing hypothesis that, if correct, would have implications for the way in which glucose levels should be managed. Little clinical data are available to directly link glucose excursions into hyper- and hypoglycemia with diabetic complications, with initial data based on self-monitoring of blood glucose suggesting no reproducible link between short-term glycemic fluctuations and development of diabetic complications [10–12]. However, new continuous glucose monitoring techniques should more fully capture the extent of glycemic fluctuation and allow firmer conclusions to be drawn. One challenge is that no gold standard exists for how glucose variability should be quantified [10]. Some groups have recently tried to develop useable indices for glycemic variability so that this question may be better studied [13–15]. We anticipate that ongoing studies should generate insight into the role of glycemic variability on the development and progression of complications [16].

Beyond management of glycemic control, new therapies are required to treat those millions of individuals already diagnosed with diabetic complications. One challenge in identifying and validating useful therapeutic targets is the chronic and multifactorial nature of diabetic complications. Hyperglycemia directly impacts multiple pathways, but as the disease progresses, additional indirect pathways are affected that may not respond quickly to improved glycemic control. Over time, as tissues attempt to regenerate and repair the damage caused by diabetes, the indirect pathways that are affected may exacerbate and complicate the overall pathology. For example, indirect pathways can promote fibrosis or vascular leak in the retina of an individual with diabetes who perhaps has “silent” manifestations of the early stages of diabetic retinopathy. Some advances have been made in the area of diabetic retinopathy, where anti-VEGF inhibitors have shown promising clinical results in treating diabetic macular edema. However, for complications including diabetic nephropathy, diabetic neuropathy, and diabetic cardiomyopathy, no specific disease-modifying therapies are widely approved or available for people with diabetes.

The generation of a robust pipeline of therapies for diabetic complications begins with the funding of innovative, early-stage research and the training of scientists and clinician–scientists in this area. Recent technological advances in genetics, transcriptomics, proteomics, and metabolomics, as well as novel approaches to analyze and exploit these data, offer a new, high-content approach to understanding diabetic complications. As the financial and time costs of these approaches decrease, we have an opportunity for a fresh look at our clinical populations. The assembly of large clinical collections with accompanying genetic information and other biological samples has enormous potential to increase our understanding of diabetic complications and to reveal new potential targets for therapy. Well-characterized clinical genetic cohorts are available, in particular for diabetic nephropathy, though early case–control studies have revealed few loci to date, perhaps due to insufficient statistical power and heterogeneity within cohorts. Larger sample sizes for genome-wide association studies and novel approaches to study design, such as examining the rate of decline of glomerular filtration rate, should be considered in order to maximize the chance of discovering new loci. The generation of genome-wide data for large and well-characterized clinical collections offers an unprecedented opportunity to look broadly at the genetic basis of other complications, such as diabetic retinopathy. However, other factors such as duration of diabetes and management of glycemic control are important in order to weigh the relative contribution of genetic and environmental factors, as discussed in the review by Paterson [17]. Better understanding of genetics could also lead to individualized therapy, and a potential paradigm for this is outlined in the review by Costacou and Levy [18], where a haptoglobin genotype may not only impact risk of diabetic complications, but also potentially the response to certain kinds of treatment.

Such clinical cohorts may also be exploited using multiple other techniques to identify potential therapeutic targets and biomarkers in diabetes complications. Improved techniques for proteomics and other analyses are yielding interesting targets in diabetic kidney disease [19], and novel tools are being developed to better mine and use such information. Integrating information from genetics, transcriptomics, proteomics, and metabolomics will be critical in building a comprehensive picture of how complications are manifested. The burgeoning field of systems biology may offer insights on the molecular pathogenesis of chronic complications, an example of which is given in the review by Kretzler and colleagues [20]. There are, however, multiple challenges to the successful exploitation of such collections. Careful assessment should be considered such as that data and samples collected decades apart may reflect very different environmental influences—particularly in glycemic control—and sample collection, preparation, and storage are rarely standardized between collections. Careful definition of phenotypes is vital to proper interpretation of data, and unfortunately, the same clinical parameters are not always measured, nor are they measured in the same way among different clinical cohorts. Efforts to harmonize data would be invaluable in the effort to understand diabetic complications. Increased communication and collaboration among investigators with these clinical cohorts, as well as wider availability of samples to others with promising hypotheses and assays, should benefit the entire research community.

Multiple mechanisms have been proposed in the development of diabetic complications. Some of these are organ specific, but several pathways may be relevant to more than one micro- or macrovascular complication of diabetes [21]. Mechanisms such as oxidative stress are the focus of intense study in diabetic complications, for example NADPH oxidase and its isoforms [22] and in some cases are being translated to clinical evaluation. One example is the initiation of clinical trials of bardoxolone for diabetic nephropathy [23]. More recently, an inhibitor of Nox1/4 [24] with potential for the treatment of diabetic nephropathy has entered phase I clinical testing. Such mechanisms are of particular interest as they may have the potential to impact multiple organ systems involved in the complications of diabetes. Understanding which pathways and targets are most important in the development of complications and would be most amenable to targeting is a current critical area of research.

Some emerging areas of research at an earlier stage of exploration include epigenetic mechanisms such as miRNAs [25] and other persistent transcriptional changes that may underlie the phenomenon known as metabolic memory; the persistent increased incidence of microvascular complications in the arm Diabetes Control and Complications Trial originally assigned to standard therapy, even many years after all participants switched to a regimen of intensive glycemic control [26]. Since genetics has thus far poorly explained the differential risk to complications, differences in these gene expression modulating pathways may help to account for additional differences in individual susceptibility to complications. A better understanding of the complex response to diabetes and the impact of persistent hyperglycemia may also reveal additional therapeutic targets.

Given the chronic nature of diabetic complications, generating faithful, predictive animal models is challenging. Generally, the most commonly used models, including streptozotocin induction of diabetes in rodents, db/db, and Akita mouse, generally reproduce only very early changes associated with complications such as retinopathy, neuropathy, and nephropathy and, in general, additional genetic mutations or other interventions, such as Western diet or uni-nephrectomy, are needed to generate a more aggressive pathology. The development of diabetic complications appears to be a stepwise process. As discussed earlier, early changes due to hyperglycemia may not mirror later changes present at the time of complication diagnosis; therefore, careful interpretation of animal model data is critical. Most published work uses rodent models in which no attempt is made to treat diabetes, generating a yet wider gap between the clinical situation and the models employed. This may account, in part, for the poor success to date in translating basic research findings into therapies for diabetes complications. The use of multiple models to mirror different aspects of disease may help alleviate this issue. For example, in testing potential agents for diabetic nephropathy, models of hypertension, fibrosis, and diabetes are available and could be employed. Encouraging basic scientists to perform animal and cell experiments, to the extent possible, in a blinded manner could increase rigor and help avoid subjective interpretation of data.

Reproducibility of findings among labs and between academia and industry is extremely poor, with one company recently reporting that they were unable to reproduce data on at least 64 % of targets identified through academic publication in peer-reviewed journals [27]. Incentives to publish confirmatory, negative, or contradictory findings are critical to translating novel findings into clinical studies and reducing the large rates of attrition in clinical trials. Facilitating the dissemination of *all* results will allow the scientific community an access to a panoramic view of the available data to support or refute a potential therapeutic target or biomarker. In the case of human clinical trials, publication of findings, whether positive or negative, is critical, considering that data generated have been generated from precious trial participants. Organizations that fund research with a focus on disease should consider the value in supporting the reproducibility of findings both to maximize the chance of translation and to eliminate the least-promising options. Opportunities for collaboration between academia and industry at earlier stages could help inform experimental design to maximize the potential for translation of research findings. Many pharmaceutical companies are now experimenting with this model, and there is also a role for research funding organizations and professional societies to promote these interactions.

The major challenge in translating exciting findings in the field of diabetic complications lies in the difficulty of conducting clinical trials. Modern standard of care and management has delayed onset and slowed progression of microvascular complications, meaning that trial durations of several years may be needed to reach critical endpoints such as change in visual acuity for diabetic eye disease or doubling time in serum creatinine, dialysis, or death for diabetic nephropathy. An additional barrier is the great heterogeneity among people with diabetes. It is inevitable that personalized therapy will be required to successfully prevent and treat diabetes complications. Biomarkers are needed to predict onset and progression of complications, to stratify patients for clinical trials, and to predict drug response at the earliest possible stage. Pharmacogenomics offers some intriguing possibilities, including the recent observation that a polymorphism in haptoglobin may impact susceptibility to complications as well as response to certain types of therapy [18].

Careful study of clinical populations to better select patients for clinical trials is clearly warranted, in particular clinical populations in which longitudinal follow-up may allow for discovery of predictive biomarkers for complication development and progression. Access to data from prior industry trials would be particularly valuable to this endeavor. A placebo effect is common in diabetes complication trials, where improvement or stabilization occurs in complications endpoints, likely due to more rigorous standard of care while in the clinical trial. This means that the placebo group of large industry or academic clinical intervention trials will likely give more accurate information on potential progression within a trial than an observational cohort study. Initiatives bringing together the communities of industry and academia, such as the IMI-SUMMIT (http://www.imi-summit.eu/) and the Biomarkers Consortium of the Foundation of the National Institutes of Health [28], offer the possibility to discover and validate such biomarkers for diabetic complications. One example of success in this area was the pooling of data from multiple trials to demonstrate the utility of adiponectin to predict decreases in HbA1c resulting from treatment of T2D [29]. These programs also contribute to a much-needed dialogue between academia and industry to help ensure translation of promising findings to impact patient care. Creative approaches to phase II clinical trial design would benefit from the discovery of biomarkers that can reliably predict longer term endpoints. In diabetic retinopathy, sensitive tests that can detect and correlate structure–function changes may hold promise, particularly given the incomplete correlation between structural measures, such as measurement of retinal swelling by optical coherence tomography, and functional measures, such as visual acuity. In order to validate such endpoints, incorporation of them as exploratory endpoints in clinical trials will be important.

To improve patient outcomes in the area of diabetic complications, it is important not only to reduce the incidence of diabetes through better treatments, prevention, or an ultimate cure, but also to have a successful continuum between innovative discovery science, rigorous translation of findings, and a better understanding of how to conduct clinical trials. A better dialogue between academia and industry is emerging, but it will take a much larger and concerted effort between academic, clinical, pharmaceutical, funding, and regulatory agencies to overcome the multiple challenges, including a lack of appropriate animal models; paucity of predictive diagnostic and functional biomarkers; lack of a proven pathway for the design clinical trials; and prohibitive length and cost of clinical trials. Government and nongovernment funders of medical research must find ways of lowering the barriers for industry entry to this highly prevalent and devastating class of diseases by better defining disease pathology and developing appropriate biomarkers for risk, onset, and progression of disease. A robust pipeline of potential therapies and targets is also vital to clinical translation, and this will depend in part upon a better understanding in the academic community of what constitutes a viable drug target and what validation is most helpful in predicting therapeutic response in human disease. Publication of negative results, both clinical and preclinical, should be encouraged. One final challenge is that although some aspects of pathology and general challenges to clinical development are common among organ systems in diabetes complications, most discussion in this area happens at organ-specific professional meetings. Smaller meetings focused on more than one diabetic complication, such as the recent Keystone Symposium on Complications of Diabetes, are to be commended for promoting a dialogue in this area of research and facilitating novel conversation and collaborations between researchers in academia, industry, and disease-specific research foundations that provide funding opportunities in this area.
